# Advanced Photocatalytic Materials

**DOI:** 10.3390/ma13040821

**Published:** 2020-02-11

**Authors:** Vlassis Likodimos

**Affiliations:** Section of Condensed Matter Physics, Department of Physics, National and Kapodistrian University of Athens, 15784 Panepistimiopolis, Greece; vlikodimos@phys.uoa.gr; Tel.: +30-2107-276-824

**Keywords:** TiO_2_ nanomaterials, visible light activated titania, heterojunction photocatalysts, photonic crystal catalysts, plasmonic photocatalysis, graphene-based photocatalysts, water and air purification, solar fuels

## Abstract

Semiconductor photocatalysts have attracted a great amount of multidiscipline research due to their distinctive potential for solar-to-chemical-energy conversion applications, ranging from water and air purification to hydrogen and chemical fuel production. This unique diversity of photoinduced applications has spurred major research efforts on the rational design and development of photocatalytic materials with tailored structural, morphological, and optoelectronic properties in order to promote solar light harvesting and alleviate photogenerated electron-hole recombination and the concomitant low quantum efficiency. This book presents a collection of original research articles on advanced photocatalytic materials synthesized by novel fabrication approaches and/or appropriate modifications that improve their performance for target photocatalytic applications such as water (cyanobacterial toxins, antibiotics, phenols, and dyes) and air (NO_x_ and volatile organic compounds) pollutant degradation, hydrogen evolution, and hydrogen peroxide production by photoelectrochemical cells.

Semiconductor photocatalysis has been considered as a key technology to face the global concerns of environmental pollution and the ever increasing energy demands, through the utilization of environmentally benign earth-abundant materials and renewable energy sources, such as solar energy [1]. Photocatalytic materials have been attracting significant interest for diverse applications, ranging from sustainable water/air remediation as well as hydrogen and chemical fuel production by photocatalytic water splitting [2]. Their unique potential for solar powered technologies has been the stimulus for the development of nanostructured photocatalysts with improved structural, morphological, and electronic properties that could effectively evade the two main limitations of the process efficiency, i.e., the low quantum yield, stemming from the recombination of photogenerated charge carriers, and the poor visible light harvesting, pertinent mostly to wide band gap semiconductors such as the benchmark titanium dioxide (TiO_2_) photocatalysts [3].

Research efforts have been accordingly focused on the design and fabrication of advanced photocatalytic materials relying on competent modification approaches such as coupling with plasmonic nanoparticles, surface engineering, and heterostructuring with other semiconducting and/or graphene-based nanomaterials, as well as tailoring the materials’ structure and morphology (e.g., nanotubes, nanowires, and photonic crystals) in order to boost light harvesting and photon capture, charge separation, and mass transfer that play a pivotal role in photocatalytic environmental remediation and solar to chemical energy conversion applications [4,5].

This Special Issue consists of 10 original full-length articles on advanced photocatalytic materials fabricated by innovative synthetic routes and judicious compositional modifications, with diverse applications ranging from the degradation of hazardous water and air pollutants to hydrogen evolution and photoelectrocatalytic hydrogen peroxide production. T. M. Khedr at al. [6] reported on the degradation of microcystin-LR (MC-LR), a highly toxic and persistent hepatotoxin commonly detected in cyanobacterial algae blooms, by visible-light-activated C/N-co-modified mesoporous anatase/brookite TiO_2_ photocatalysts, prepared by a one-pot hydrothermal method. The complete removal of MC-LR from aqueous solutions was achieved under visible light irradiation, related to the unique combination of visible light photogenerated electrons from anion-induced impurity states and interfacial charge transfer between the brookite and anatase phases.

N-modified TiO_2_ was utilized by M. Janus et al. [7] as an additive for the technologically appealing application of photocatalytic active cement mortars that feature air purification by NO_x_ decomposition without compromising, and even improving, cement’s mechanical properties. Laser pyrolysis was applied by K. Wang et al. [8] to synthesize novel C-modified titania/graphene nanocomposites with markedly high activity on different photocatalytic applications from acetic acid oxidative decomposition and methanol dehydrogenation (even without a Pt co-catalyst) to visible-light induced phenol degradation and Escherichia coli inactivation. Surface functionalization of TiO_2_ photonic crystals by graphene oxide (GO) nanocolloids and subsequent thermal reduction was reported by Diamantopoulou et al. [9] as a promising approach for the development of efficient photocatalytic films that combine the unique slow photon-assisted light harvesting, surface area, and mass transport of macroporous photonic structures with the enhanced adsorption capability, surface reactivity, and charge separation of GO nanosheets.

Fluorine-doped tin oxide (FTO) inverse opals crystal were also exploited by X. Ke et al. [10] as macroporous photonic crystal substrates for the successive deposition of plate-like WO_3_ and Ag_2_S quantum dots with tunable photoelectrochemical response by the light incidence angle. Control over the shape and facet growth of TiO_2_ nanocrystals was demonstrated by Y. Du et al. [11] by tuning the pH of the exfoliated metatitanic nanosheet solutions used as precursors in a simple fluorine-free microwave-assisted hydrothermal method, leading to enhanced photocatalytic and photovoltaic performance. The oriented morphology and enhanced surface area of TiO_2_ nanowires, grown on TiO_2_ nanotube arrays by electrochemical anodization, in combination with Au plasmonic nanoparticles were successfully applied by T.C.M.V. Do et al. [12] for the photocatalytic degradation of eight important antibiotics in model aquaculture wastewater from the Mekong Delta region.

Plasmonic Ag nanomaterials were also incorporated in silver-copper oxide heterostructures by H. Suarez et al. [13] to promote the full-spectrum photocatalytic-assisted volatile organic compound (VOC) oxidation in the gas phase, using n-hexane as a probe molecule, with LED illumination in the visible-NIR range. V. H. Nguyen et al. [14] reported on sulfate modification as an efficient means to improve the structural and photocatalytic properties of sol-gel synthesized BiVO_4_ with annealing temperature, used as an alternative metal oxide photocatalyst beyond TiO_2_, leading to enhanced decomposition of methylene blue as a model dye pollutant under LED visible light.

The sensitization of mesoporous Titania films by nanoparticulate CdS and CdSe, in combination with ZnS passivation was used by T. S. Andrade et al. [15] for the fabrication of ZnS/CdSe/CdS/TiO_2_/FTO photoanodes, enabling broad visible light harvesting. These photoelectrodes combined with a Pt-free counter electrode, made of carbon cloth with deposited nanoparticulate carbon, were used for the assembly of a photoelectrochemical cell operating as a Photo Fuel Cell, i.e., without any external bias. These devices were demonstrated to photoelectrocatalytically produce substantial quantities of hydrogen peroxide with the Faradaic efficiency exceeding 100% in the presence of NaHCO_3_ carbonate electrolyte.

All the published articles were thoroughly refereed through the standard single-blind peer-review process of Materials journal. As Guest Editor, I would like to acknowledge all of the authors for their excellent contributions and the reviewers for their valuable and prompt comments that greatly improved the quality of the papers. Finally, I would like to thank the staff members of Materials, in particular Ms. Floria Liu, Section Managing Editor, for her devoted efforts and kind assistance.
